# Transit From Autotrophism to Heterotrophism: Sequence Variation and Evolution of Chloroplast Genomes in Orobanchaceae Species

**DOI:** 10.3389/fgene.2020.542017

**Published:** 2020-10-06

**Authors:** Ruiting Zhang, Bei Xu, Jianfang Li, Zhe Zhao, Jie Han, Yunjing Lei, Qian Yang, Fangfang Peng, Zhan-Lin Liu

**Affiliations:** Key Laboratory of Resource Biology and Biotechnology in Western China (Ministry of Education), College of Life Science, Northwest University, Xi’an, China

**Keywords:** Orobanchaceae, plastomes, hemiparasites, gene loss, selection, phylogeny

## Abstract

The family Orobanchaceae including autotrophic, hemiparasitic, and holoparasitic species, is becoming a key taxa to study the evolution of chloroplast genomes in different lifestyles. But the early evolutionary trajectory in the transit from autotrophism to hemiparasitism still maintains unclear for the inadequate sampling. In this study, we compared 50 complete chloroplast genomes in Orobanchaceae, containing four newly sequenced plastomes from hemiparasitic *Pedicularis*, to elucidate the sequence variation patterns in the evolution of plastomes. Contrasted to the sequence and structural hypervariabilities in holoparasites, hemiparasitic plastomes exhibited high similarity to those of autotrophs in gene and GC contents. They are generally characterized with functional or physical loss of *ndh*/tRNA genes and the inverted small-single-copy region. Gene losses in Orobanchaceae were lineage-specific and convergent, possibly related to structural reconfiguration and expansion/contraction of the inverted region. Pseudogenization of *ndh* genes was unique in hemiparasites. At least in *Pedicularis*, the *ndhF* gene might be most sensitive to the environmental factors and easily pseudogenized when autotrophs transit to hemiparasites. And the changes in gene contents and structural variation potentially deeply rely on the feeding type. Selective pressure, together with mutational bias, was the dominant factor of shaping the codon usage patterns. The relaxed selective constraint, potentially with genome-based GC conversion (gBGC) and preferential codon usage, drive the fluctuation of GC contents among taxa with different lifestyles. Phylogenetic analysis in Orobanchaceae supported that parasitic species were single-originated while holoparasites were multiple-originated. Overall, the comparison of plastomes provided a good opportunity to understand the evolution process in Orobanchaceae with different lifestyles.

## Introduction

Chloroplasts are the key organelles for green plants, where light energy can be converted into chemical energy through photosynthesis. Chloroplast DNAs of land plants have widely been sequenced and typically have highly conserved structure with a large and a small single-copy region (LSC and SSC, respectively) separated from each other by two identical inverted repeat (IR) regions (Jansen and Ruhlman, 2012). Generally, chloroplast genomes (plastomes) range in size from 120 to 160 kb with 120–130 genes, many of which encode proteins, tRNAs, and rRNAs essential for photosynthesis (Williams et al., 2015). Research works on plastomes can provide valuable information not only to reconstruct the phylogenetic relationships among complex taxa due to their high resolution (Huang et al., 2014), but also to understand the evolutionary history of plants with different lifestyles. For non-photosynthetic plants retrieving nutrients partially or exclusively from the hosts, photosynthesis is not the only process to obtain energy. Genes involved in photosynthesis are no longer required and free from selective constraints. Therefore, the plastomes in heterotrophs have undergone various changes in genome size and structure, such as pseudogenization, gene loss and genome rearrangements (Cusimano and Wicke, 2016; Graham et al., 2017). For example, the plastomes of heterotrophic species in Ericaceae are highly reduced in size and have lost all photosynthesis-related genes (Braukmann et al., 2017). Large-scale structural reconfigurations have occurred in the holoparasitic species of Orobanchaceae (Wicke et al., 2016). Loss of IRs and *ndh* genes and accelerated substitution rates of protein-coding genes were reported in a hemiparasitic herb *Cassytha filiformis* (Wu et al., 2017).

The broomrape family, Orobanchaceae, contains about 90 genera and more than 2000 species with autotrophic, hemiparasitic and holoparasitic lifestyles, becoming a model for tracing the evolutionary trajectories of non-photosynthetic plant genomes (Westwood et al., 2012). Except for the non-parasitic genera of *Lindenbergia*, *Triaenophora*, and *Rehmannia*, the rest can be classified as holoparasites or hemiparasites (Byng et al., 2016). Hemiparasites have green leaves and are capable of photosynthesis while holoparasites without photosynthetic tissue depend on their hosts for all their requirements. Hemiparasites include two groups: facultative and obligate. Contrasting to the obligate, facultative hemiparasites can fulfill its lifecycle without a host. Phylogenetic works supported that parasitism in Orobanchaceae was single originated and there were at least three times of the origin in holoparasitic taxa (Samigullin et al., 2016). Recently, the evolution of plastomes in Orobanchaceae species was investigated (Wicke et al., 2011, 2013) and hypothesis was proposed that stepwise gene loss followed by the functional loss of photosynthesis was convergent in the degradation route of plastomes (Wicke and Naumann, 2018). At the beginning of heterotrophs, non-essential or stress-relevant genes (*ndh*) are non-functional or lost, followed by primary photosynthesis-related genes (*pet*, *psa*), and non-essential housekeeping genes in the process of plastome degradation. When photosynthesis-unrelated metabolic genes and other housekeeping genes are deleted, the degradation reaches its final stage (Wicke and Naumann, 2018). But the evolutionary hypothesis was conducted by plastome comparisons mainly between autotrophs and holoparasites. The reduction of plastomes might be diverse (Liu et al., 2020) and lineage-specific (Wicke et al., 2013). As the early stage from autotrophs to heterotrophs, the evolutionary trajectories of hemiparasitic plastomes are still not well-known. More hemiparasitic species should be necessarily involved to investigate the evolutionary patterns of plastomes.

*Pedicularis* is the largest lineage of photosynthetic root hemiparasitic plants with about 600 species, widely distributed in the northern temperate zone (Yu et al., 2015). Most of species in the genus are facultative and narrowly distributed. The diversified morphological characters made classification of the genus still unresolved (Yu et al., 2015). Research works are mainly focused on its phylogenetic position and interspecific relationships. Interestingly, *Pedicularis* species are considered as primitive parasites with very wide host ranges. Majority of species in the genus are potentially both parasitic and mycorrhizal. Some could live well without dominant hosts in artificial condition (Li and Guan, 2008). The slight dependence on hosts made *Pedicularis* a good model for heterotrophism. Studies on plastomes in the genus potentially shed new lights on variation pattern at the first stage of genome evolution in Orobanchaceae. In this study, we newly sequenced four *Pedicularis* plastomes, and performed comparative genome analysis with their autotrophic or holoparasitic relatives, together with 13 publicly available hemiparasitic plastome data in Orobanchaceae. Our aims were mainly to characterize the hemiparasitic plastomes, to uncover the sequence divergence and structural changes, and investigate the early stage of the evolutionary trajectories of plastomes in Orobanchaceae with variable lifestyles.

## Materials and Methods

### Sample Collection, Sequencing, Genome Assembly, and Annotation

Four *Pedicularis* species were collected from their natural habitats [*P. resupinata* L. (voucher No. 15-0327), *P. muscicola* Maxim. (voucher No. 15-0345), *P. oederi* Vahl (voucher No. 15-0381), 107.71°E, 34.03°N; *P. longiflora* Rudolph (voucher No. 16-0012), 91.10°E, 30.78°N]. All specimen were deposited in Herbarium of Northwest University. Next generation sequencing was constructed by Illumina Hiseq 2500 platform in Biomarker Technologies, Inc. (Beijing, China). Raw reads were quality-trimmed using the NGS QC Toolkit v2.3.3 (Patel and Jain, 2012) allowing a maximum of 2% ambiguous base calls. The clean reads were mapped to the plastome sequence of *Castilleja paramensis* (KT959111) using the software MITObim v1.8 with the default parameters (Hahn et al., 2013). The assembled contigs were generated into a consensus sequence in GENEIOUS R8 (Biomatters, Ltd., Auckland, New Zealand), then annotated using the program DOGMA (Wyman et al., 2004) and manually corrected by comparing them with the reference plastome in GENEIOUS R8 program.

### Sequence Divergence and Rearrangement Analysis

To identify the sequence divergence of autotrophic, hemiparasitic, and holoparasitic species of Orobanchaceae, four new *Pedicularis* plastomes, together with the 46 publicly available cpDNA sequence from GenBank, including 8 autotrophs, 13 hemiparasitic species and 25 holoparasitic species, were used in the evolutionary analyses. All taxa in this study are listed in the Supplementary Table S1 with their GenBank accession numbers. Sequences were aligned using MAFFT (Katoh and Standley, 2013) in GENEIOUS R8 with the default parameters set and manually edited when necessary. Sequence divergence was investigated with DnaSP v5 (Librado and Rozas, 2009) and MEGA v7.0.18 (Kumar et al., 2016). Sliding window analysis was conducted to generate nucleotide diversity (π) using program DnaSP. The step size was set to 200 bp with a window length of 600 bp. Mauve Alignment (Darling et al., 2004) was performed to reveal the rearrangement among plastome of these species.

### Codon Usage Analysis

The program CodonW (Sharp and Li, 1987)^1^ was employed for the analysis of synonymous codon usage in the protein coding regions (>300 bp). We tabulated two codon usage measures: the effective number of codons (Nc) and frequency of the GC at the third synonymous position (GC3s) (Ivanova et al., 2017). Estimation of the standard effective number of codon (ENc) was tabulated using the equation ENc = 2 + s + 29/[s^2^ + (1 – s)^2^], where s denoted GC3s (Wright, 1990). We also calculated ENc ratio (ENc_exp_-ENc_obs_)/ENc_exp_, to estimate the difference between observed and expected ENc values (Kawabe and Miyashita, 2003).

### Selection Pressure Analysis

To calculate the non-synonymous (*dN*) and synonymous (*dS*) substitution rates and their ratio (ω = *dN/dS*), the nine protein-coding genes shared in 50 Orobanchaceae species were extracted and aligned separately. The value of *dN*, *dS* and ω for each protein-coding gene were calculated using site-specific model implemented in the codeml package (seqtype = 1, model = 0, NSsites = 1, 2, 7, 8) of PAML v4.7 (Yang, 2007), holding the maximum likelihood (ML) tree constructed with plastomes by using software RAxML (Stamatakis, 2014). Adaptive evolution of each gene was determined by computing likelihood ratio tests (LRTs). To check the presence of positively selected sites, we examined the LRTs for positive selection of two pairwise models: (1) M1a (Nearly Neutral; ω*_0_* < 1, ω*_1_* = 1) against M2a (Positive Selection; ω*_0_* < 1, ω*_1_* = 1, ω*_2_* > 1), while (2) M7 (Neutral; β) against M8 (Selection; β and ω). The Bayes empirical Bayes (BEB) procedure was used to identify the positively selected sites with the degree of freedom (*df*) of two. Only candidate sites of positive selection with significant support values from posterior probability (ω > 1; *p* ≥ 0.99; BEB) identified by M2 and M8 were considered further (Nielsen and Yang, 1998; Yang and Nielsen, 2002).

We used RELAX (Wertheim et al., 2015), which is available from the Datamonkey webserver^2^, to test the changes in the relative selection intensity on all most shared genes in Orobanchaceae. Given test and reference branches in a codon-based phylogenetic framework, RELAX can calculate a selection intensity parameter value (*k*) and its statistical significance, with *k* > 1 showing intensified selection and *k* < 1 indicating relaxed selection.

### Phylogenetic Analyses

Plastomes of fifty representative species in Orobanchaceae were used for the reconstruction of phylogenetic tree with two Lamiaceae species, *Tectona grandis* (NC_020431) and *Rosmarinus officinalis* (NC_027259), as outgroup. Because of the highly variable molecular evolutionary rates among the different plastome regions, phylogenetic relationship analyses were performed using nine conserved protein-coding genes (*rps2*, *rps4*, *rps7*, *rps8*, *rps14*, *rpl2*, *rpl16*, *rpl36*, and *matK*) in Orobanchaceae. We determined the best-fitting evolutionary model using Modeltest v3.7 (Posada and Crandall, 1998) with the Akaike Information Standard (AIC) score (Posada and Buckley, 2004). PartitionFinder 2 (Lanfear et al., 2017) was used to estimate optimal partitioning schemes by grouping similar data blocks from the output of the remaining datasets. Phylogenetic analysis with maximum likelihood (ML) method was carried out using software RAxML v8.2 (Stamatakis, 2014) with 1000 bootstrap replicates. Bayesian inference (BI) analyses were performed with program MrBayes v3.1.2 (Ronquist and Huelsenbeck, 2003). The Markov chain Monte Carlo (MCMC) algorithm was run for 10,000,000 generations with trees sampled every 10,000 generations for each data partition. The first 25% of trees from all runs were discarded as burn-in, and the remaining trees were used to construct the majority-rule consensus tree. FigTree v.1.31 was used to visualize the tree topology (Rambaut, 2006). The phylogenetic relationships among hemiparasitic species in Orobanchaceae were further identified by using the ML method with 55 common intact genes.

## Results

### Chloroplast Genome Structure of Four *Pedicularis* Species

**FIGURE 1:**
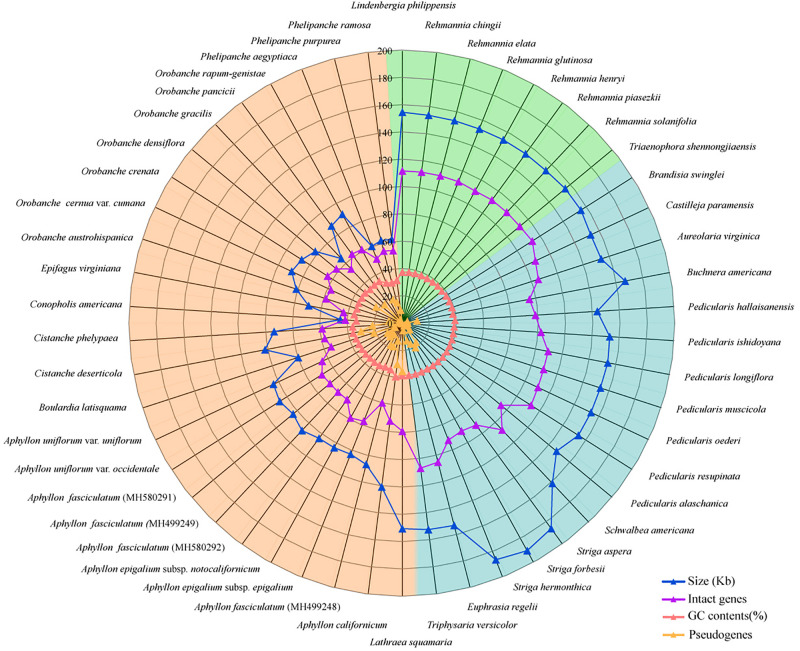
A radar-plot for comparing features of the complete chloroplast genomes in 50 Orobanchaceae species. From inside to outside: pseudogenes, GC contents, number of protein-coding genes, and genome sizes. The background colors of green, blue, and orange represent autotrophic, hemiparasitic, and holoparasitic species, respectively.

Illumina sequencing technology produced sufficient data (with coverage > 150X) for plastome assembly from 17,462,980 (*P. muscicola*) to 23,876,956 (*P. oederi*) paired-end reads. The plastomes of four *Pedicularis* species sequenced here (GenBank accession numbers: MH703578 ∼ MH703581) had a typical quadripartite structure with high similarity in sizes (from 152,907 bp in *P. muscicola* to 153,547 bp in *P. longiflora*) (Supplementary Table S1 and Figure 1). They also have the identical GC contents (about 38%) and good collinear structure (not shown here). Each *Pedicularis* plastome contained 133 genes, including 37 tRNA genes and eight rRNA genes, of which 115 occurred as single copy genes and 18 genes were duplicated in the IR regions (Supplementary Tables S1, S2). The number of protein-coding genes (PCGs) and pseudogenes varied from species to species. There were 88 PCGs and no pseudogene in *P. resupinata*. In contrast, two pseudogenes (*ndhD* and *ndhF*) existed in *P. oederi*, and three pseudogenes (*ndhD*, *ndhF*, and *ndhH*) were found in *P. muscicola* and *P. longiflora* (Supplementary Table S2). These pseudogenes had multiple predicted frameshift mutations and premature stop codons located at different sites. We compared IR/LSC and IR/SSC junctions in our *Pedicularis* plastomes and found that two copies of *ycf1* gene spanned the SSC/IR junctions, while the *ndhF* gene crossed the SSC region into IRa region with some variable nucleotides in *Pedicularis* species, except *P. resupinata* (Supplementary Figure S1). Contrasting to the relatively stable SC/IR boundaries in the four newly sequenced species, plastomes of *P. alaschanica*, *P. hallaisanensis* had prominent shifts of SSC/IRa boundaries due to loss of *ndh* genes (see below) and *P. ishidoyana* had the shortest SSC region (27 bp) for the expansion of IRs (Supplementary Figure S1).

### General Features of Chloroplast Genomes in Orobanchaceae

A total of 50 plastomes of Orobanchaceae were utilized to analyze the variation of sequence and structure, including 8 autotrophs, 17 hemiparasites, and 25 holoparasites species (Supplementary Table S1 and Figure 1). The largest plastome was 190 kb (*Striga forbesii*), about 4.2 fold larger than the smallest plastome of *Conopholis americana* (46 kb). Autotrophs and hemiparasites generally have similar genome sizes, varying from 143 kb (*P. hallaisanensis*) to 160 kb (*Schwalbea americana*) with unexpected larger plastomes in *Striga* species. A significant reduction of genome sizes was observed in the holoparasites plastomes with a range from 46 kb (*Conopholis americana*) to 150 kb (*Lathraea squamaria*). The GC contents of plastomes also dramatically decreased in holoparasites (35.0% in average), in contrast to the slightly but significantly different GC values in hemiparasites (38.2% in average) and autotrophs (37.9% in average) (Supplementary Figure S2).

**FIGURE 2:**
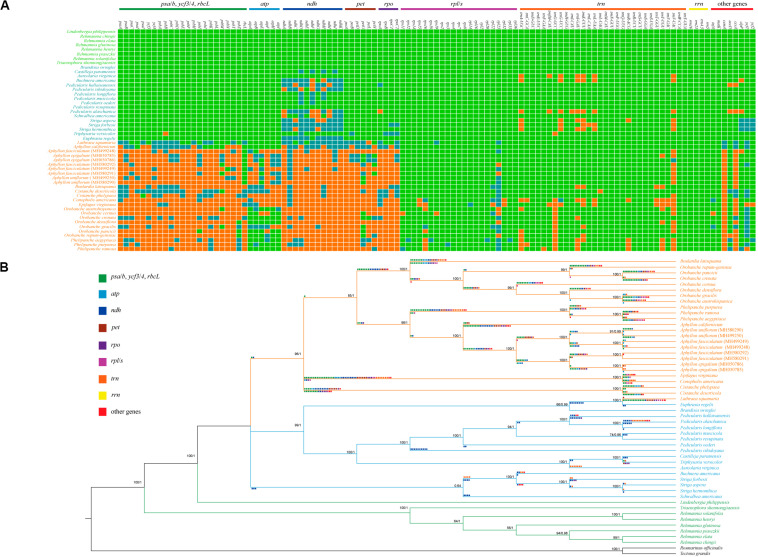
Relationships between gene contents and phylogenetic tree of Orobanchaceae plastomes. **(A)** Heat map of the presence or absence of chloroplast genes in autotrophs (species names in green), hemiparasites (species names in blue), and holoparasites (species names in orange). Green blocks, presence of a gene; blue, presence of a pseudogene; orange, absence of the gene from the chloroplast genome. **(B)** Inferred gene losses in the phylogenetic tree of Orobanchaceae reconstructed using ML and BI methods. Pseudogenization is illustrated below the branches, whereas gene deletion is shown above the branches.

**FIGURE 3:**
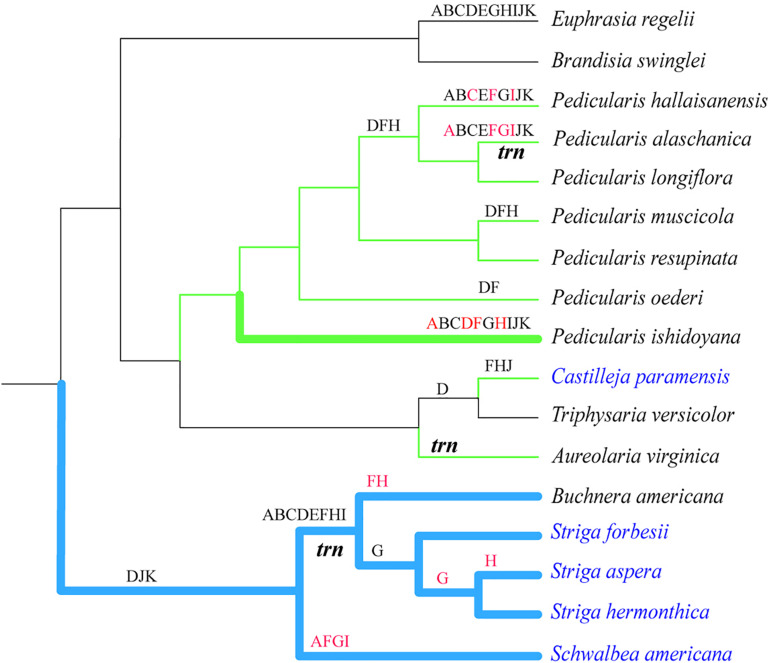
Relationships among phylogeny, lifestyles, structural rearrangements, and gene non-functionalization in hemiparasitic plastomes. ML tree was constructed by using 55 conserved intact genes. Thicken branches represented lineages with the expanded IR region. Blue and green branches showed the lineages with the rearrangement of LSC/SSC and the inverted SSC regions, respectively. Black and red letters above branches represented the pseudogenized and missing *ndh* genes, respectively. The italic *trn* indicated the majority of tRNA genes were lost in the lineages. Taxa in black and blue were the facultative and obligate hemiparasites, respectively.

When functional genes simply counted, only 26 genes, including nine PCGs (*rps2*, *rps4*, *rps7*, *rps8*, *rps14*, *rpl2*, *rpl16*, *rpl36*, and *matK*), four rRNAs genes, and 13 tRNA genes were commonly present in all 50 plastomes of Orobanchaceae species (Figure 2A). Comprising 133 genes, autotrophs had no gene loss and pseudogenes. Pseudogenization and loss of the *ndh* genes were commonly observed in the hemiparasitic plants. The functional or physical deletion of tRNA gene family, generally together with other housekeeping genes, is unique at the genus level (Figure 3). For holoparasites, further gene loss and non-functionalization were commonly found with most of the photosynthetic genes, such as *psa/psb* (photosystem I and II), *pet* (cytochrome b6/f complex), *ccsA* (heme attachment factor), *cemA* (inner membrane protein for CO2 uptake), *ycf3/4* (both photosystem assembly factors), and *ndh* (Figure 2A). Phylogenetic tree of the 50 Orobanchaceae species reconstructed by all most conserved plastome genes indicated that gene loss was highly lineage-specific (Figures 2B, 3).

### Sequence Divergence and Rearrangement in Orobanchaceae Plastomes

Sequence divergence estimated by nucleotide diversity showed that sequence variation increased from the autotrophs (π = 0.0147) to heterotrophs (the holoparasites had the highest, π = 0.1430) (Supplementary Table S3). This tendency was also confirmed by sliding window analysis (Supplementary Figure S3). The autotrophic plants held relatively conserved plastomes (π = 0∼0.069), of which *psbE-petL*, were the most variable region (Supplementary Figure S3A); For the hemi-parasitic plants, the range of nucleotide diversity was much wider (π = 0.0025∼0.4177) with the most variable region of the *ndh* gene cluster (*ndhH*, *ndhA*, *ndhI*, *ndhG*, and *ndhE*) (Supplementary Figure S3B); Due to excessively alignment gaps in plastomes of holoparasitic species, net number of nucleotide substitution per site was limited and sliding window analysis could not be successfully performed. This result indicated that contrast to the low level of variation in the most parts of plastomes of autotrophs and hemiparasites, all regions of the holoparasitic plastomes harbored high variation of nucleotide acids.

Linear rearrangement comparisons across the plastome sequences (one IR region removed) of 50 Orobanchaceae species were conducted by aligning with *Lindenbergia philippensis* as the reference. All autotrophs presented the completely identical collinear plastomes (data not shown). By contrast, heterotrophs exhibited lineage-specific structural variation from good collinearity to high reconfiguration. Among the hemiparasitic species, plastomes in *Brandisia swinglei* and *Triphysaria versicolor* had similar gene orders to autotrophs while others had partly or completely inverted and rearranged SSC regions to a variable extent (Supplementary Figure S4A). In contrast to the stability of the LSC and IR regions in most hemiparasites, a 5 kb inversion was found in the LSC region of *Schwalbea americana* and frequent large-scale structural reconfigurations existed in LSCs of *Buchnera americana* and three *Striga* species. In holoparasitic plants, plastomes of *Lathraea squamaria* and species in *Aphyllon* genus showed a good collinearity to autotrophs with genome reduction and occasionally inverted sequences in SSC. But high structural reconfigurations, such as inversion, a large scale of reduction or even loss of the IR region were usually found in the majority of holoparasitic taxa (Supplementary Figure S4B).

### Codon Usage and Selection Events in Protein-Coding Genes

A notable tendency of high A/T contents, especial the third codon position (GC3s, with the mean value of 0.25), was commonly found in all plastomes (Supplementary Table S1 and Supplementary Figure S4B). The effective number of codons (ENc) is widely used to measure the codon bias. The ENc values varied from 33 to 61, demonstrating a significant difference in the codon usage bias (CUB) with extreme bias or equal usage among genes in Orobanchaceae plastomes. Genes in hemiparasites usually presented the highest values of GC3s and ENc while holoparasites had the lowest (Supplementary Figure S4B), indicating the stronger preference of codon usage of AT in holoparasitic cp genes. The ENc-plot distribution (ENc versus GC3s) showed that ENc values of most genes were far away from the expected ENc curve, suggesting that these genes were not solely constrained by the compositional constraints (Supplementary Figure S5) and other factors (e.g., translational selection) might influence the features of codon usage. The possibility of natural selection shaping the codon usage of the photosynthesis genes (*psa/b*, *ycf3/4*, *rbcL*) was also supported by their narrow distribution of GC3s (Kawabe and Miyashita, 2003). Genes below or above the ENC curve are likely under positive or negative selection pressure for codon usage (Wright, 1990). Therefore, these photosynthesis genes in autotrophs and hemiparasites had potentially experienced a selection in opposite direction for codon usage. The frequency distribution of ENc ratios showed that there was a simple peak of −0.05∼0.15 in most chloroplast genes, except the *ndh* genes in holoparasites. The major genes of heterotrophic species usually had ENc ratio values of −0.05∼0.05. By contrast, autotrophs harbored higher ENc ratio values (0.10–0.15) in core photosynthesis genes (*pet*) and ATP synthase (*atp*) genes, suggesting that they had slightly smaller ENc values than expected, and other factors apart from compositional constrain, such as selection pressure, influenced CUB as implied by ENc plots (data not shown).

To identify genes under the positive selection, nine protein-coding genes shared among 50 Orobanchaceae species were extracted to calculate the *dN/dS* ratio (ω) using KaKs_calculator (Zhang et al., 2006). The *dN/dS* ratios of *rps2*, *rps14*, and *rpl36* gene were almost above 1.0 in all 50 species, indicating that these genes were potentially under the pressure of positive selection in Orobanchaceae (Supplementary Figure S6). The genes of *rps4*, *rps7*, *rps8*, *rpl2*, *rpl16*, and *matK* were subjected to purifying selection (*dN/dS* < 1). When the site-specific selection events were identified by using the *dN/dS* analysis, both LRTs models (M1 vs. M2; M7 vs. M8) supported *rps2* gene had potentially undergone the positive selection with one selective site (Supplementary Table S4).

We used the codon-based phylogenetic framework RELAX to test the change of selection pressure. Among the shared nine genes of Orobanchaceae species with different lifestyles, a significantly relaxed selection intensity of *rpl2* gene was found between autotrophs and heterotrophs (*k* = 0.45, *p* < 0.01). There was no significant relaxed or intensified selection existing between hemiparasites and holoparasites (*p* = 0.86).

### Phylogenetic Analysis

The phylogenetic tree based on nine shared genes in Orobanchaceae with ML and BI methods had an identical topological structure with moderate-to-high support values. The monophyletic Orobanchaceae was well-supported (Figure 2B). The non-parasitic taxa of *Triaenophora* and *Rehmannia* formed a basal lineage and the third autotrophic taxon, *Lindenbergia philippensis*, was sister to all other parasite species as supported in the previous study (Wicke et al., 2013). The holoparasitic *Lathraea squamaria* was clustered with hemiparasites, demonstrating the multiple origins of the holoparasites (Samigullin et al., 2016). All hemiparasitic species were grouped into two major lineages. *Schwalbea* was close to *Striga* and *Buchnera*, but with weak support values. The genus *Pedicularis* was monophyletic and sister to *Castilleja*-*Triphysaria*-*Aureolaria* clade. Interspecific relationships within *Pedicularis* were also determined and the clearly basal position of *P. ishidoyana* was identified in both ML and BI phylogenetic trees (Figures 2B, 3).

## Discussion

### Plastome Size and Structure Rearrangement

Parasitic species in Orobanchaceae were proven to be a monophyletic group (Ree, 2005; Yu et al., 2015) and became a good model for the plastome evolutionary study. In this work, we compared the complete chloroplast genomes of 50 species to understand the evolutionary transition in the early stage from autotrophs to holoparasites. Plants in the family exhibited a high variation of plastomes in size and structure. Genome reduction and multiple rearrangements mainly occurred in the holoparasitic species (Figure 1 and Supplementary Figure S4B) and well-discussed (Wicke et al., 2013; Wicke and Naumann, 2018). In Santalales, hemiparasites generally characterized by the reduction of chloroplast genome (Li et al., 2017; Shin and Lee, 2018; Wicke and Naumann, 2018). But most of the hemiparasitic plastomes in Orobanchaceae were similar to those of autotrophs in genome size, GC and gene contents (Figure 1 and Supplementary Table S1) (Wicke et al., 2013; Zeng et al., 2017). This stable structure indicated their plastomes were just at the very early stage of transition from autotrophs to holoparasites (Wicke and Naumann, 2018). Slight plastome reduction were observed in *P. hallaisanensis* and *P. alaschanica*, which was essentially due to the loss of *ndh* genes (Figure 2 and Supplementary Figure S4). By contrast, recent works in *Buchnera americana* and three *Striga* species uncovered an unexpected enlargement of plastomes due to the expansion of the inverted repeats into the single copy region (Frailey et al., 2018). This increase of plastome size was rather rare and its effects were still unknown.

All plastomes of hemiparasites in this study had a typical quadripartite structure with slight expansions and contractions in the IR/SC boundary region with an exception as mentioned above. Structural reconfiguration of plastomes was gradually increased and lineage specific. *Brandisia swinglei*, *Triphysaria versicolor*, and *Euphrasia regelii* had no large rearrangement compared to the autotrophic plastomes. All these hemiparasites are facultative and heavily depend on photosynthesis. No or a small scale of rearrangement is expected in these species (Frailey et al., 2018). Sequence inversions and rearrangements in the SSC region were the common feature in the rest hemiparasitic plastomes (Supplementary Figure S4A). These large inversions have also been reported in other angiosperms, such as Asteraceae (Jansen and Palmer, 1987) and Leguminosae (Jansen et al., 2008). The chloroplast genome was hypothesized with a flip-flop recombination and two equally chloroplast structural haplotypes occurred in most land plant individuals (Wang and Lanfear, 2019). But the phenomenon that nearly all hemiparasites shared the inverted SSC might not be simply ascribed to the random capture of two haplotypes when sequence reads were assembled. And further studies are necessarily needed to identify its universality. In addition to the inverted SSC region, the clade of *Schwalbea*-*Buchnera*-*Striga* also had inversion and rearrangements of LSC fragments (Frailey et al., 2018), which implied their specificity in the phylogenetic position and the evolutionary trajectory of plastomes.

### Pseudogenization and Gene Losses

From autotrophs to heterotrophs, the genome size and the gene number in plastomes decreased gradually along with the increasing number of the pseudogenization and gene loss (Figure 1). The reduction is mainly due to the loss of genes on a variable scale. Pseudogenes and gene losses were not found in autotrophs of Orobanchaceae, but appeared occasionally in most of hemiparasites, and became rather common in holoparasites (Figure 2) (Wicke et al., 2016). Several models of the plastome degradation have been proposed to explain the physical or functional change linked with the transition to heterotrophy. *Ndh* genes are often the first non-functional or lost genes due to parasitism, followed by the photosynthesis-related and -unrelated apparatus. The housekeeping genes maintain functional till the final stage of the chloroplast reduction (Barrett and Davis, 2012; Barrett et al., 2014; Naumann et al., 2016; Wicke et al., 2016; Graham et al., 2017; Wicke and Naumann, 2018). Our data well-supported the hypothesis. Holoparasitic species in Orobanchaceae exhibited serious functional or physical gene loss including photosynthesis genes (*atp*, *ndh*, *psa/b*, *ycf3/4*, *rbcL*, *pet*, *ccsA*, *cemA*), housekeeping genes (*rpo*, *rpl/s*), and tRNA genes. By contrast, some hemiparasitic *Pedicularis* species harbored none or a few *ndh* pseudogenes (Figure 2), indicating the earliest stage of parasites.

*Ndh* genes are composed of 11 genes (*ndhA* ∼ *ndhK*), encoding the thylakoid NAD(P)H dehydrogenase complex together with the nuclear genes *ndhL* ∼ *ndhO.* The complex plays a critically important role in photosynthesis. Meanwhile, their mutation rates are high and sensitive to environmental conditions and stress (Silva et al., 2016). For heterotrophs, once the parasitic system was set, the mode of carbon uptake and utilization changed and *ndh* genes became dispensable. The process in the functional loss of *ndh* genes is still unknown. In *Pedicularis*, the number of pseudogenized or lost *ndh* genes varied from none (*P. resupinata*) to all (*P. hallaisanensis* and *P. alaschanica*) (Figure 3). According to the hypothesis of the degradation of plastomes in a stepwise manner, dispensable *ndh* genes should be pseudogenized successfully before they were physically deleted (Wicke et al., 2016; Wicke and Naumann, 2018). Pseudogenes could be generated by premature stop codons and frameshift mutations (Balakirev and Ayala, 2003; Poliseno et al., 2010). In the newly studied *Pedicularis* species, *ndh* pseudogenes were verified for the presence of several internal stop codons. Among the absent member of *ndh* gene family, *ndhD* and *ndhF* were pseudogenized or lost in all seven *Pedicularis* species except *P. resupinata* (Figure 3). The non-functionalization in *Pedicularis* plastome might have opportunities to star with the pseudogenization of *ndhD* and *ndhF* genes. The clues are also found in other parasitic groups. These two genes are also identified as commonly inactive genes in the lineage of *Striga* (Frailey et al., 2018). The losses of *ndhF* are ancestrally shared in the Santalales (Shin and Lee, 2018). The *ndhF* gene is generally located in the junction of SSC/IRa (Supplementary Figure S1). The frequent contraction and expansion of the IRs, a common evolutionary event in angiosperms (Kode et al., 2005), might affect the stability of IRs and their immediate neighborhood, leading to the increase of mutation rates and further loss of the neighbor *ndh* genes (Wicke et al., 2011; Li et al., 2017). The inversion and rearrangement of the SSC region in hemiparasites could potentially accelerate this procedure (Supplementary Figure S4). In reverse, the loss of the *ndh* genes is likely to lead to the structural changes of plastomes. Contrasted to species with intact or pseudogenized *ndh* genes, those with lost *ndh* genes had undergone a slight shift of IR/SSC (*P. hallaisanensis* and *P. alaschanica*) or expansion IRs (*P. ishidoyana*) (Figure S4). The loss of *ndh* genes, especially *ndhF*, might play every important role in the shift of the IR/SC boundary of plastomes (Kim et al., 2015; Cho et al., 2018). But the assumption of pseudogenization starting with *ndhF* in *Pedicularis* should be tested further in a wide range of heterotrophs. More works, especially functional and physiological analysis, are necessarily needed in future to uncover the evolutionary trajectories of *ndh* genes.

In heterotrophs, gene loss is tightly linked to the dependent extents of parasites on the hosts (Cusimano and Wicke, 2016; Wicke and Naumann, 2018). Their different reliance to the host potentially shaped the evolution of chloroplast genome to some extent (Barrett et al., 2014; Naumann et al., 2016; Wicke et al., 2016; Wicke and Naumann, 2018). Although *Pedicularis* is commonly considered as the root hemiparasitic group, little is known about its parasitic habit. The recent study uncovering the coexistence of haustorial and mycoheterotrophic parasites demonstrated that *Pedicularis* had complex nutrient uptake strategy (Li and Guan, 2008). Unfortunately, the parasitic habit of *Pedicularis* species involved in this study retains unknown. Justified from no or quite a few pseudogenes, these *Pedicularis* species might slightly rely on the host plants. But the functional and/or physical loss of genes is not determined by heterotrophy *per se* (Wicke et al., 2011, 2014), i.e., facultative hemiparasites might not necessarily harbor less non-functional genes than the obligate. Structural variation as mentioned above could also affect the evolution of *ndh* genes. For example, *P. ishidoyana* had much more lost or pseudogenized *ndh* genes than most of the facultative species (Figure 3).

The multiple origins of the facultative species were found in Orobanchaceae as verified in orchids (Barrett and Davis, 2012), carnivorous plants (Silva et al., 2016), even gymnosperms (Lin et al., 2010). In the monophyletic genus of *Pedicularis* (Ree, 2005; Robart et al., 2015), species are phylogenetically independent to non-functionalization or loss genes of *ndh*. For instance, in the phylogenetic tree, *P. ishidoyana*, a basal lineage in *Pedicularis*, lost all *ndh* genes (except *ndhE*), while *P. resupinata* contained all functional *ndh* genes (Figure 3). These results showed that parallel pseudogenization or loss of *ndh* genes frequently occurred in *Pedicularis*, as reported in previous works (Frailey et al., 2018; Shin and Lee, 2018). When phylogenetic relationships were separately analyzed with each gene, the loss was derived directly from the intact gene or indirectly from the pseudogene as proposed by Liu et al. (2020).

Contrasting to *Pedicularis*, the clade of *Schwalbea*-*Buchnera*-*Striga* exhibited lineage-related pattern of gene loss. Most *ndh* genes are lost early in the lineage, but *ndhG* and *ndhH* are only absent in the terminal branches, indicating *ndh* genes sequences might retain despite the loss of function (Frailey et al., 2018). In addition, *Buchnera*-*Striga* clade also lost several tRNA genes. This degradation pattern is associated with rearrangement of SSC/LSC and contraction/expansion of IR regions (Figure 3). The basal *Schwalbea* had the lowest structural variation and gene loss while the terminal *Striga* showed the highest (Figures 2, 3 and Supplementary Figure S4). This group would potentially be a key lineage for analysis of the gradual degradation pattern in plastome evolution. This phylogeny-related pattern of gene loss was also found in Santalales. Genes of *ndhF*, *ndhI*, and *ndhJ* are commonly absent in all Santalales species. *NdhA*, *ndhC*, *ndhD*, *ndhE*, *ndhG*, *ndhH*, and *ndhK* are lost in *Viscum* of Viscaceae (Shin and Lee, 2018).

### Codon Usage, GC Content, and Selective Pressure

Variable rates of molecular evolution are often associated with life history in heterotrophs (Smith and Donoghue, 2008). With the transition to a parasitic lifestyle, the chloroplast genome will experience relaxed selection pressure, accelerated evolutionary rate, and reduced GC contents during the evolution process (Wicke et al., 2016; Wu et al., 2017; Shin and Lee, 2018; Wicke and Naumann, 2018). According to our results, holoparasites exhibited higher structural variation and lower GC contents in plastomes in contrast to their autotrophic relatives while hemiparasitic plants harbored similar or slightly higher GC contents than autotrophs (Figure 1 and Supplementary Figure S2). One hypothesis is that these changes in heterotrophs are random byproducts of transition of lifestyle because plastomes are not directly involved in the parasite/host interaction (Wicke and Naumann, 2018). The alternative is that along with the reduction of genome size, especially the loss of genes with higher GC contents, the decrease of GC contents is becoming a general trait in heterotrophic plastomes (Wicke et al., 2013). The lowest GC contents in holoparasitic plastome could be well-explained by this hypothesis. However, unexpected slightly high GC values of hemiparasitic plastomes, mainly in LSC, were found in our study (Supplementary Figure S2A). This was not simply ascribed to the sampling bias of limited autotrophs involved in the study when a generally low GC value (<38%) was found in autotrophs of the relative families (Phrymaceae and Paulowniaceae). The slightly increased GC contents were closely linked to the inverted SSC in hemiparasites. The exteriorly poor GC contents in the *Schwalbea-Buchnera-Striga* clade were ascribed to the low GC values in IRs (Supplementary Table S1). IR is a hotspot region of mutation and rearrangement. Frequent contraction/expansion makes a generally AT-riched IR (Wicke et al., 2013) and produces an unstable structure in plastome. The process of GC-based gene conversion (gBGC) prefers repairing DNA mismatches with GC bases and elevating GC contents over evolutionary time (Niu et al., 2017). It shapes both the plastome-wide mutations and GC contents as a complementary mechanism for maintaining the structure and function of plastome with rearranged sequences.

CUB toward AT driven by ultraviolet-radiation and DNA methylation was found in all Orobanchaceae species, also commonly in the chloroplast genomes of land plants (Ossowski et al., 2010; Qi et al., 2015). CUB was primarily determined by the dynamic balance associated with mutations and natural selection, as well as the structure and functions of genes, gene expression, and many other factors in the evolutionary process (Bulmer, 1991; Chiapello et al., 1998; Wong et al., 2002). In our study, the scattered distribution of ENc values in many genes, far from the expected curves, would not be ascribed to mutation pressure alone, implying other factors, for example, natural selection might have effects on CUB as reported in many plants (Liu and Xue, 2005) (Supplementary Figure S5). This selective pressure is considered to promote the translation efficiency for the chloroplast genomes (Suzuki and Morton, 2016; Zhang et al., 2018). Most genes in autotrophic plastomes were possibly driven by selective pressures to adapt the efficient functional express in the process of photosynthesis. But in heterotrophs, the changes in the mode of nutrients acquisition relaxed the selective constraints and compositional mutation of GC to AT transition (Krasovec and Filatov, 2019) became the main factor shaping CUB, finally resulting in the prominent decrease of the GC3s usage as observed in holoparasitic plastomes and supported by the non-significant shift of ENc ratios (data not shown). In our study, we observed an unexpected slight higher GC3s in hemiparasites than those in autotrophs. This finding was associated with the potentially negative or positive selection for photosynthesis genes in autotrophic and hemiparasitic plastomes, respectively (Supplementary Figure S5). We hypothesized that along with the change of lifestyles, the opposite selection in hemiparasites perhaps drove a shift of CUB toward GC. The changes in the direction of selection for codon usage was also found diatoms (Krasovec and Filatov, 2019). But it is still unknown that to what extent, the interaction of mutation pressure and natural selection influence on the patterns of codon usage. Therefore, this hypothesis of the preferential codon usage under natural selection should be further investigated with more measures. An alternative was that the increase of GC3 was a balance of mutation pressure and CUB toward AT. As discussed above in GC content variation, gBGC might elevate GC contents across the whole plastome and possibly counteracted the effect of CUB toward AT. Because no mutation rate and gBGC were examined here, the hypothesis needs to test in future.

Relaxed selective constraint was usually considered as the main factor driving gene more divergent in heterotrophs than in autotrophs (Wicke et al., 2011). The codon-based phylogenetic framework was used to test the selective pressure between autotrophic and heterotrophic plastomes in Orobanchaceae. We uncovered that *rpl2* gene had undergone relaxed selection, indicating an early accelerated base substitution rate along with the evolutionary history of heterotrophs in the lineage (Wicke et al., 2011). The gene *rpl2* located in the LSC/IRb junction and might be affected by the expansion/contraction of IR. And the relaxed purifying selection is potentially linked to the lowered photosynthesis rate in heterotrophs (Wicke et al., 2014). We analyzed the selective pressures of the functional genes retained in all Orobanchaceae species and identified three genes (*rps2*, *rps14*, and *rpl36*) were subject to positive selection and only *rps2* gene had positive selection sites (Supplementary Table S6 and Supplementary Figure S6). These genes encode the large or small subunit ribosomal protein together with other genes to ensure the process of initiation of protein synthesis. The genes of *rps2* and *rps14* are essential components for the plastid translation. Despite the non-essential component for ribosome, the loss of *rpl36* would cause a severe mutation phenotype (Tiller and Bock, 2014). Consequently, these genes (especially *rps2*) under natural selection play an important role in the evolution and divergence of Orobanchaceae.

### Phylogenetic Relationships

Phylogenetic trees of Orobanchaceae using chloroplast or ITS gene fragments indicated that there was a deep split between heterotrophic and autotrophic plants, i.e., parasitic species were monophyletic (Olmstead et al., 2001). Our analysis based on plastomes drew a similar conclusion (Figure 2). The holoparasitic *Lathraea squamaria* is always clustered with hemiparasites, suggesting the multiple origins of holoparasitic species (Young et al., 1999; Olmstead et al., 2001; McNeal et al., 2013). *Pedicularis* is monophyletic and related to the clade of *Aureolaria*-*Triphysaria*-*Castilleja* (Figures 2, 3). This is not completely consistent with previous studies (Robart et al., 2015; Yu et al., 2018), mainly due to limited hemiparasitic plastomes available in our study. The future work on plastomes involving more basal branches, such as *Agalinis*, *Seymeria* and *Phtheirospermum*, will shed further lights on the evolutionary history of Orobanchaceae. As for *Pedicularis*, recent molecular phylogenetic works showed that most groups based on morphological traits were not monophyletic (Tkach et al., 2014; Robart et al., 2015; Yu et al., 2018). In this study, *P. ishidoyana* was the basal taxon, which was consistent with morphological traits and molecular data (Cho et al., 2018). But it is hard to compare the interspecific relationships of other species to the previous phylogenetic studies (Tkach et al., 2014; Yu et al., 2018) for the lack of plastomes in most *Pedicularis* species. More samples are urgently needed to clarify to phylogenetic relationships of this divergent genus in future.

## Conclusion

The changes in lifestyle could trigger the degradation of plastomes in heterotrophs. Here, we compared the plastomes of 50 Orobanchaceae species in order to elucidate the sequence variation pattern in the transition from autotrophic to holoparasitic lifestyles. Plastomes of hemiparasites exhibited much more similarities to the autotrophic relatives with a few pseudogenized/lost *ndh* and tRNA genes, suggesting that these species were just at the very early stage of transition from autotrophs to heterotrophs. Both structural rearrangement and relaxed selective constraints contributed to the changes of GC content and gene order along with the transit of lifestyles. Due to the clade specific evolution of plastomes in many aspects, more samples from the monophyletic hemiparasitic genus, such as *Pedicularis* and *Striga*, are necessarily needed to investigate the divergently evolutionary trajectories of chloroplast genomes in Orobanchaceae.

## Data Availability Statement

The datasets generated for this study can be found in the GenBank accession numbers: MH703578–MH703581.

## Author Contributions

Z-LL and RZ conceived and designed the experiments. JL, QY, and FP performed the experiments. RZ and BX analyzed the data. ZZ, JH, and YL prepared the samples. RZ wrote the manuscript. Z-LL helped to revise the manuscript. All authors read and approved the final manuscript.

## Conflict of Interest

The authors declare that the research was conducted in the absence of any commercial or financial relationships that could be construed as a potential conflict of interest.

## Footnotes

1 http://codonw.sourceforge.net/

2 https//www.datamonkey.org/RELAX

## Supplementary Material

Supplementary Figure 1 Comparison of the borders of LSC, SSC and IR regions in the plastomes of *Pedicularis* species with *Lindenbergia philippensis* as the reference. Ψ indicates a pseudogene. This figure is not to scale.

Supplementary Figure 2 GC **(A)** and GC3s **(B)** contents in different regions of plastomes. Significant test values were labeled (ns, non-significant; ^∗^*p* < 0.05, ^∗∗^*p* < 0.01, ^∗∗∗^*p* < 0.001).

Supplementary Figure 3 Sliding window analysis of the whole chloroplast genomes of autotrophs **(A)** and hemiparasites **(B)**. X-axis: position of the midpoint of a window, Y-axis: nucleotide diversity of each window.

Supplementary Figure 4 Collinearity of plastomes of autotrophs **(A)**, hemiparasites **(B)**, and holoparasites **(C)** in Orobanchaceae using the MAUVE algorithm with *Lindenbergia philippensis* as the reference. Local collinear blocks are colored in this figure to indicate syntenic regions, while histograms within each block represent the degree of sequence similarity.

Supplementary Figure 5 ENc-plots (ENc vs. GC3s) for different gene groups of 50 plastomes in Orobanchaceae.

Supplementary Figure 6 The *dN/dS* ratio of *rpl36*, *rps2* and *rps14* gene in Orobanchaceae plastomes. Green – autotrophic species, blue – hemiparasitic species, and orange – holoparasitic species.

Supplementary Table 1 Chloroplast genome characteristics of 50 taxa in Orobanchaceae.

Supplementary Table 2 Gene contents of four *Pedicularis* plastomes.

Supplementary Table 3 The number of variable sites and nucleotide diversity in plastomes of Orobanchaceae.

Supplementary Table 4 Positive selection sites estimated with Maximum Likelihood parameter in *rps2* gene.
