# Supplementary figures and images for: Transit From Autotrophism to Heterotrophism: Sequence Variation and Evolution of Chloroplast Genomes in Orobanchaceae Species
Source: Front Genet. 2020 Oct 6;11:542017. doi: 10.3389/fgene.2020.542017 (PMC7573133; doi:10.3389/fgene.2020.542017)

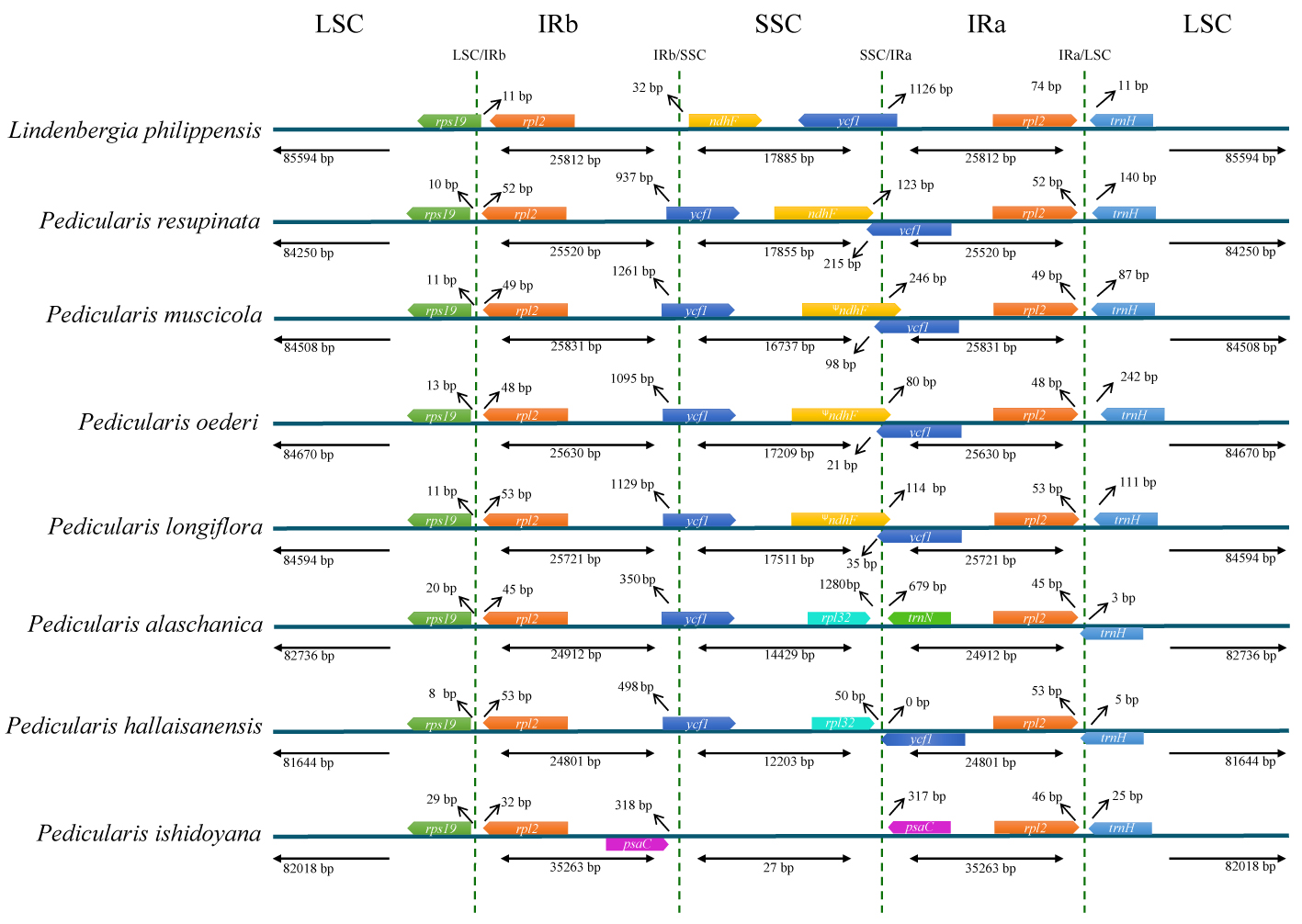

Supplement: Supplementary Figure 1 — Comparison of the borders of LSC, SSC and IR regions in the plastomes of Pedicularis species with Lindenbergia philippensis as the reference. Ψ indicates a pseudogene. This figure is not to scale. [file Image_1.JPEG]

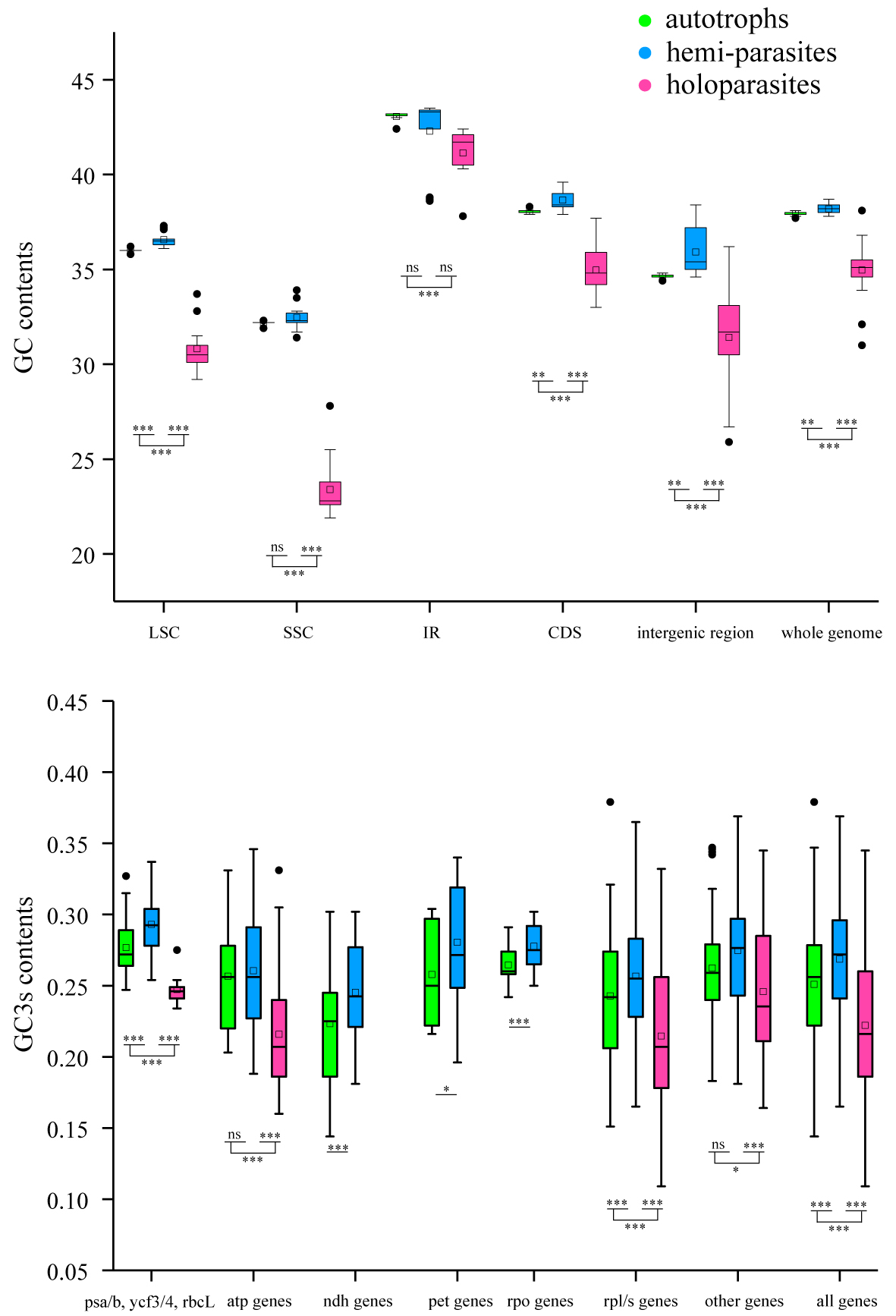

Supplement: Supplementary Figure 2 — GC (A) and GC3s (B) contents in different regions of plastomes. Significant test values were labeled (ns, non-significant; ∗p < 0.05, ∗∗p < 0.01, ∗∗∗p < 0.001). [file Image_2.JPEG]

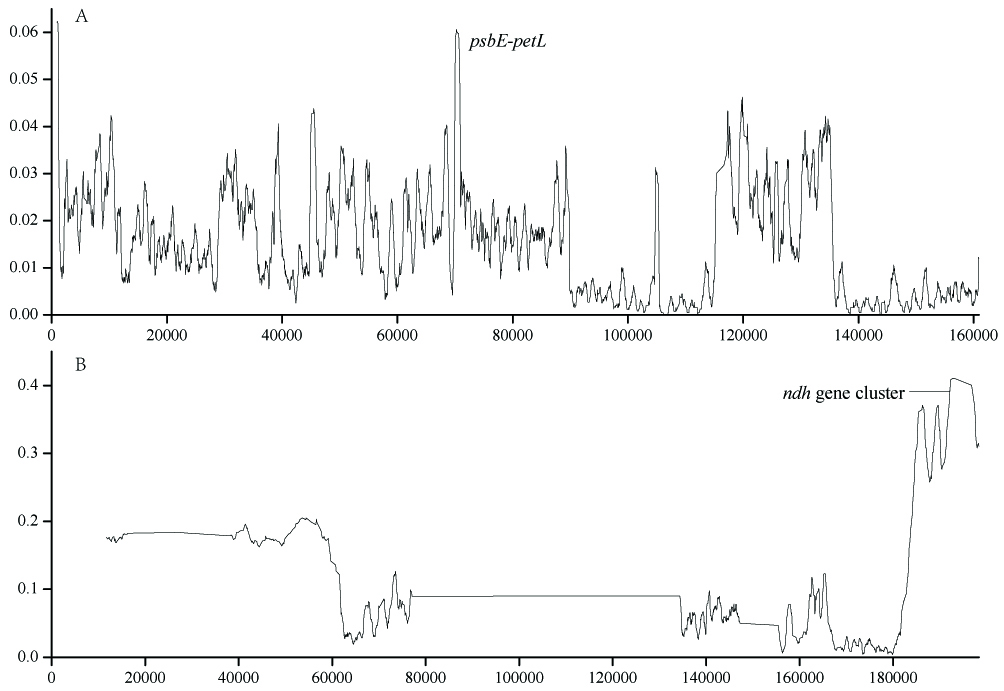

Supplement: Supplementary Figure 3 — Sliding window analysis of the whole chloroplast genomes of autotrophs (A) and hemiparasites (B). X-axis: position of the midpoint of a window, Y-axis: nucleotide diversity of each window. [file Image_3.JPEG]

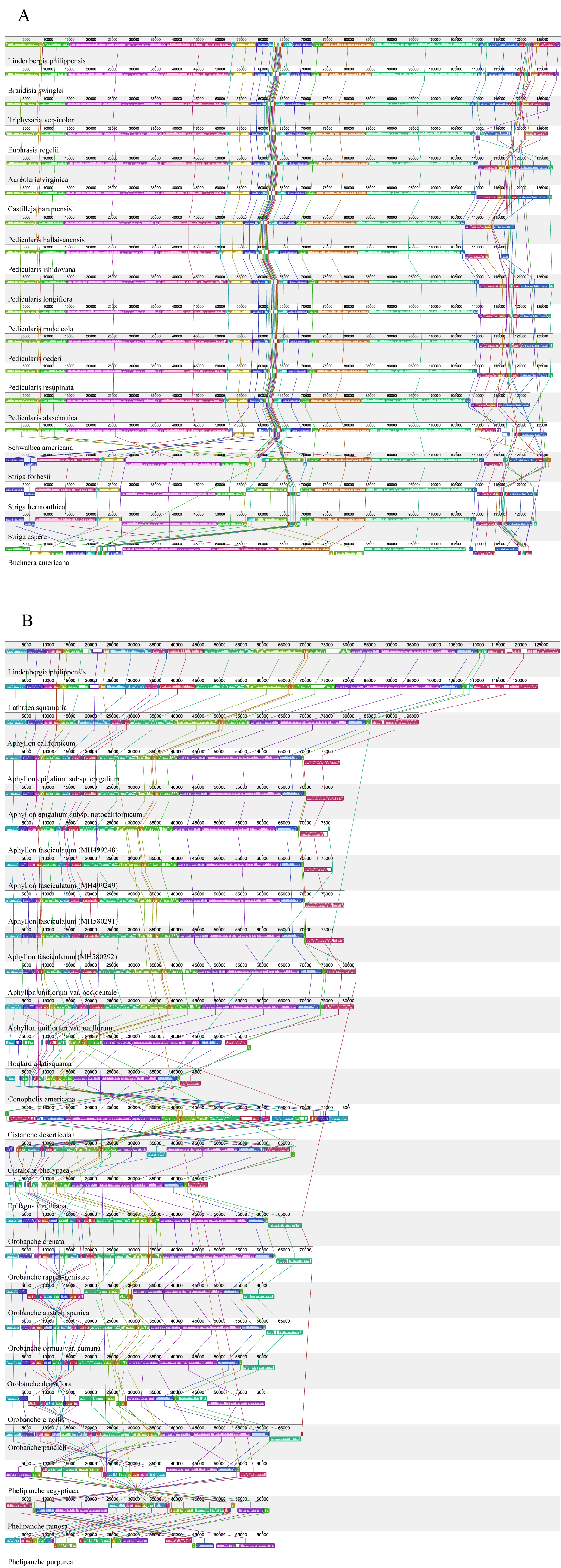

Supplement: Supplementary Figure 4 — Collinearity of plastomes of autotrophs (A), hemiparasites (B), and holoparasites (C) in Orobanchaceae using the MAUVE algorithm with Lindenbergia philippensis as the reference. Local collinear blocks are colored in this figure to indicate syntenic regions, while histograms within each block represent the degree of sequence similarity. [file Image_4.JPEG]

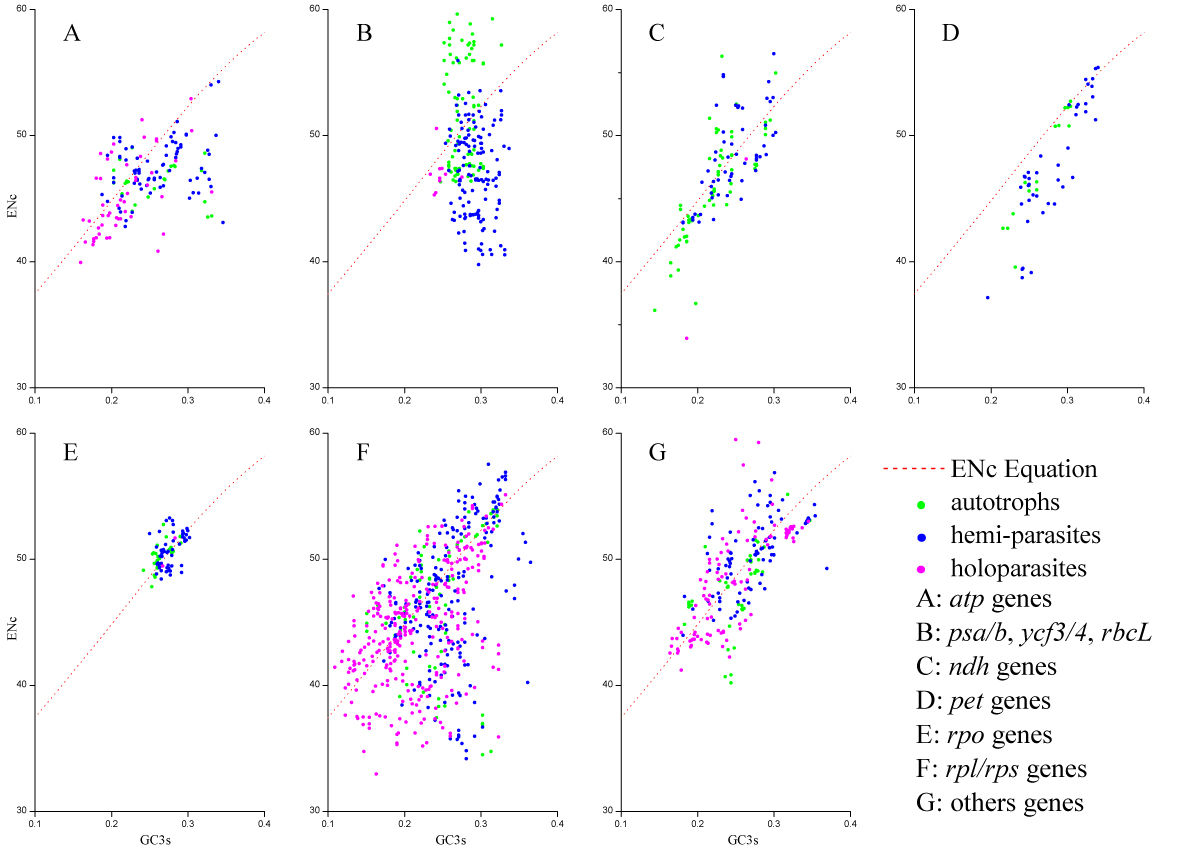

Supplement: Supplementary Figure 5 — ENc-plots (ENc vs. GC3s) for different gene groups of 50 plastomes in Orobanchaceae. [file Image_5.JPEG]

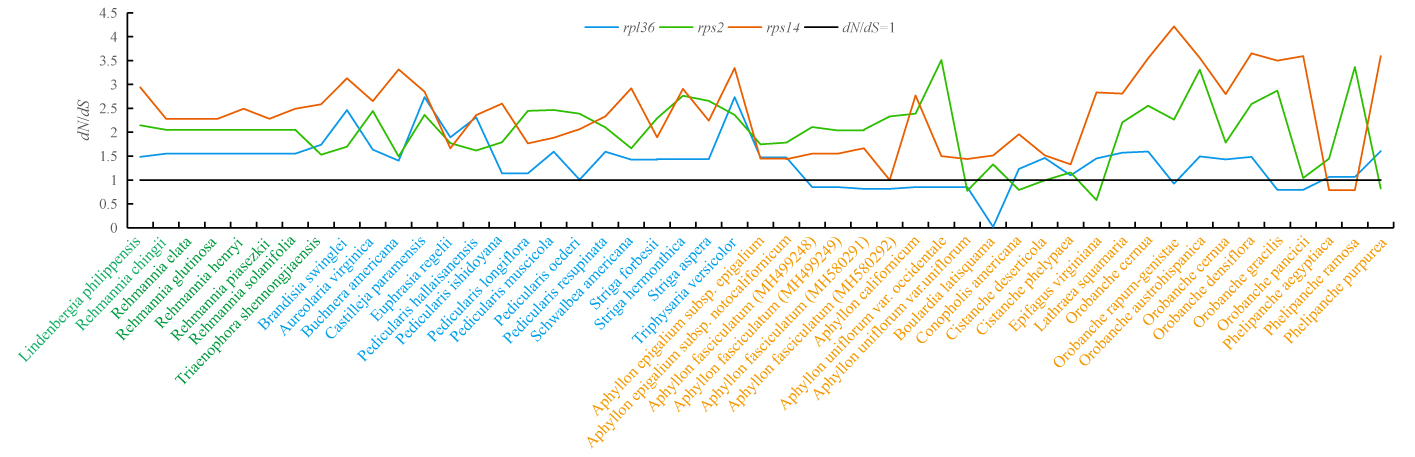

Supplement: Supplementary Figure 6 — The dN/dS ratio of rpl36, rps2 and rps14 gene in Orobanchaceae plastomes. Green – autotrophic species, blue – hemiparasitic species, and orange – holoparasitic species. [file Image_6.JPEG]
